# Functionality and quality of life in Brazilian patients 6 months post-stroke

**DOI:** 10.3389/fneur.2023.1020587

**Published:** 2023-04-20

**Authors:** Ana Railka de Souza Oliveira-Kumakura, Larissa Mariana Oliveira Santos Batista, Gabriela Salim Spagnol, Lenise Valler

**Affiliations:** ^1^School of Nursing, University of Campinas, Campinas, Brazil; ^2^Nursing Sciences Research Chair, Laboratory Education and Health Promotion, Université Sorbonne Paris Nord, Bobigny, France; ^3^Nursing School, Anhanguera University, Sumaré, Brazil; ^4^Knowledge Management and Education Solutions, Cogna Education, Valinhos, Brazil; ^5^Clinical Hospital, University of Campinas, Campinas, Brazil

**Keywords:** stroke, quality of life, functionality, rehabilitation, care

## Abstract

**Background:**

Surviving a stroke poses a social and economic impact that requires the care system to be reformulated and the patient to be addressed in a comprehensive approach.

**Purpose:**

This study aims to investigate if there is a relationship between functional activities performed before the stroke, patients' clinical and hospitalization data, and functionality and quality of life measures in the first 6 months after the stroke.

**Methods:**

This study used a prospective cohort of 92 patients. We investigated sociodemographic and clinical data, the modified Rankin Scale (mRS), and the Frenchay Activities Index (FAI) during hospitalization. The Barthel Index (BI) and EuroQol-5D (EQ-5D) were applied at the following time points: 30 days (T1), 90 days (T2), and 180 days (T3) following postictal state. Statistical analysis was conducted using Spearman's coefficient, Friedman's non-parametric test, and multiple linear regression models.

**Results:**

No correlation was found between FAI, BI, and EQ-5D average scores. Severe patients, patients with comorbidities, and patients with extended hospital stays showed lower BI and EQ-5D scores □in the follow-up. BI and EQ-5D scores increased.

**Conclusion:**

This research found no relationship between activities performed before the stroke and functionalities and quality of life after the stroke, but comorbidities and extended hospital stay were associated with worse outcomes.

## Introduction

According to 2019 data from the Global Burden of Disease, stroke is the second-leading cause of death in the world (11.6% of total deaths) and the third-leading cause of death and disability combined (5.7% of total disability-adjusted life-years) (1). In Brazil, the age-standardized mortality rate from stroke per 100,000 decreased from 137.8 in 1990 to 58.1 in 2019. This study also showed a prominent decrease in years of life lost (YLL): the age-standardized YLL rates due to stroke per 100,000 in 1990 were 2778.6 and, in 2019, 1098.7 (2).

In Brazil, healthcare expenditures for acute treatment of incident ischemic stroke amounts to a direct cost of USD 326.9 million (ranging from 82.4 to 732.2) and USD 122.4 million (ranging from 30.8 to 274.2) for intracranial hemorrhage in 2006-2007 (3, 4). Most of these costs directly impact the public financial agenda, considering that only 18% of the population in the country has access to private insurance (5).

Stroke also brings indirect costs related to loss of productivity and affects individuals and their families. In addition to its sudden health burden, stroke may compromise the ability to work, resulting in financial support needs (6, 7). Therefore, returning to work is an essential stroke-related outcome in occupations (8, 9).

Also, the concept of quality of life (QoL) has been widely applied to assess stroke's impact on one person's life. According to the World Health Organization, QoL is defined as “*perception of the individual about his position in life, in the context of the culture and system of values in which he lives, and in relation to his goals, expectations, standards, and concerns*” (10). This concept considers personal, physical, and psychological characteristics and social aspects to provide a multidimensional indicator of individual well-being and health in the exploration of daily living functions (11, 12).

Studies have applied the concept of QoL with two different approaches: a more generic perspective, without reference to dysfunction or injuries, and health-related quality of life (HRQoL) associated with diseases or health interventions (13). HRQoL evaluates the patient's perception in four dimensions: physical, functional, psychological, and social, encompassing personal beliefs (13).

Although studies present different assessment methods in different populations and different degrees of health impact, data shows that stroke significantly affects various domains of QoL (13, 14), compromising functionality (6, 15, 16). Specifically, in HRQOL, stroke survivors present lower mean scores for physical health (−7.9 %), mental health (−4.1 %), and health utility (−6.9 %) than the non-stroke population (17).

Studies show that lower QoL after stroke is associated with a lack of functional independence, depression, older age (18, 19), and cognitive impairment (20, 21). Stroke negatively affects QoL in younger (22) and older (23) cohorts, but contributing factors may differ across the lifespan. A decrease in the ability to concentrate among youth after a stroke presented a relationship with lower QoL (24). At this age, in a group of working-age survivors of a mild stroke, even minor stroke-related deficits represented an important limitation in reestablishing leisure (58%) and work activities (52%) (25). Given QoL is based on the individual's life perspectives and relates to different expectations and goals, factors such as geographic region or educational background may also influence the impact of stroke (26). In this sense, another important concept in assessing health outcomes post-stroke is functionality.

The International Classification of Functioning, Disability and Health (ICF) defines functionality as the interaction between the health condition and the individual's environment and context. Functionality relates to autonomy and independence through preserving cognition (mental ability to understand and solve everyday problems), humor (motivation for activities and/or social participation), mobility (ability to move and physically interact), and communication (ability to establish a productive relationship) (27). Regarding physical (dis)ability, functional capacity has been widely assessed according to physical function degrees of independence and dependence in Barthel's categories of daily life activities (28).

In this case, independence consists of the ability to perform functions related to daily life, and to live independently in the community with or without support to perform self-care or daily activities (29). Autonomy comprises the ability of decision-making and life self-management according to personal rules, beliefs, and ethical and moral values, free from the influence of others (30–32).

Therefore, functional capacity is the ability to maintain the physical and mental skills necessary for an independent and autonomous life. In the case of stroke, functional capacity is associated with previous health condition and environmental, socioeconomic, cultural, and personal factors that may favor a situation of functional disability (30).

The term disability encompasses impairments (loss of function) and limitations or restrictions of social participation. Disability may be classified according to degrees of dependence (total, partial or minimal) in self-care, self-preservation, and survival activities (basic activities of daily living—BADL); household chores, such as cooking or cleaning (instrumental activities of daily living—IADL); and social and recreational activities (advanced activities of daily living—AADL) based on observed or self-reported data (30).

Due to the limited access to rehabilitation services, especially in low-income communities, up to 70% of Brazilian stroke patients do not have access to rehabilitation programs (3). In Brazil, 33% of stroke survivors present an overall proportion of functional dependence (modified Rankin Scale 3 to 5) at discharge. This number decreases to 12% in 1 year, 9% in 2 years, and 8% in 3 years (33).

Variables that explain epidemiological differences in stroke incidence, severity, and functional impairment can help identify groups at increased risk. Risk factors for stroke can be classified as non-modifiable and modifiable. Non-modifiable risk factors include advanced age, male, black and Hispanic, with a family history of stroke, history of transient ischemic attack, and genetic conditions (34). Modifiable risk factors are subdivided into behavioral risk factors related to tobacco or alcohol intake, unbalanced diet, or being overweight; and potential health risk factors, as hypercholesterolemia, arterial hypertension, diabetes mellitus, metabolic syndrome, cardiovascular diseases (atrial fibrillation, acute myocardial infarction, atherosclerosis, among others), chronic kidney disease, sleep apnea, use of oral contraceptives or hormone replacement therapy, and exposition to air pollution (34).

Studies often use the mRS, the Barthel Index (BI), and the Lawton and Body Inventory to assess health outcomes after stroke. These tools can include relevant information to improve rehabilitation planning, and patients may be guided to primary and secondary prevention recommendations and healthcare in the early stages of rehabilitation, according to their specific needs (13).

Post-stroke neurological recovery peaks especially in the first three months after stroke and continues in the following three months (35). A vision of functional recovery and a global approach to QoL improvement during rehabilitation may improve stroke survivors' health outcomes (13, 36). This study aimed to investigate if there is a relationship between functional activities performed before the stroke and functionality and quality of life measures in the first six months after stroke.

## Methods

### Design

This was a single-site, longitudinal, quantitative study following the Strengthening the Reporting of Observational Studies in Epidemiology (STROBE) Statement for cohort studies (37).

### Participants

Individuals diagnosed with acute cerebrovascular event, stroke, or transient ischemic attack (TIA) during the acute/subacute phase were recruited from a public hospital in Campinas, São Paulo, Brazil, between October 2017 and May 2019. Inclusion criteria were a medical diagnosis of stroke and receiving medical assistance for the acute event in the hospital's emergency service. Exclusion criteria: patients incapable of verbal communication and with no caregivers who could provide information about the health and disease process.

**Figure 1:**
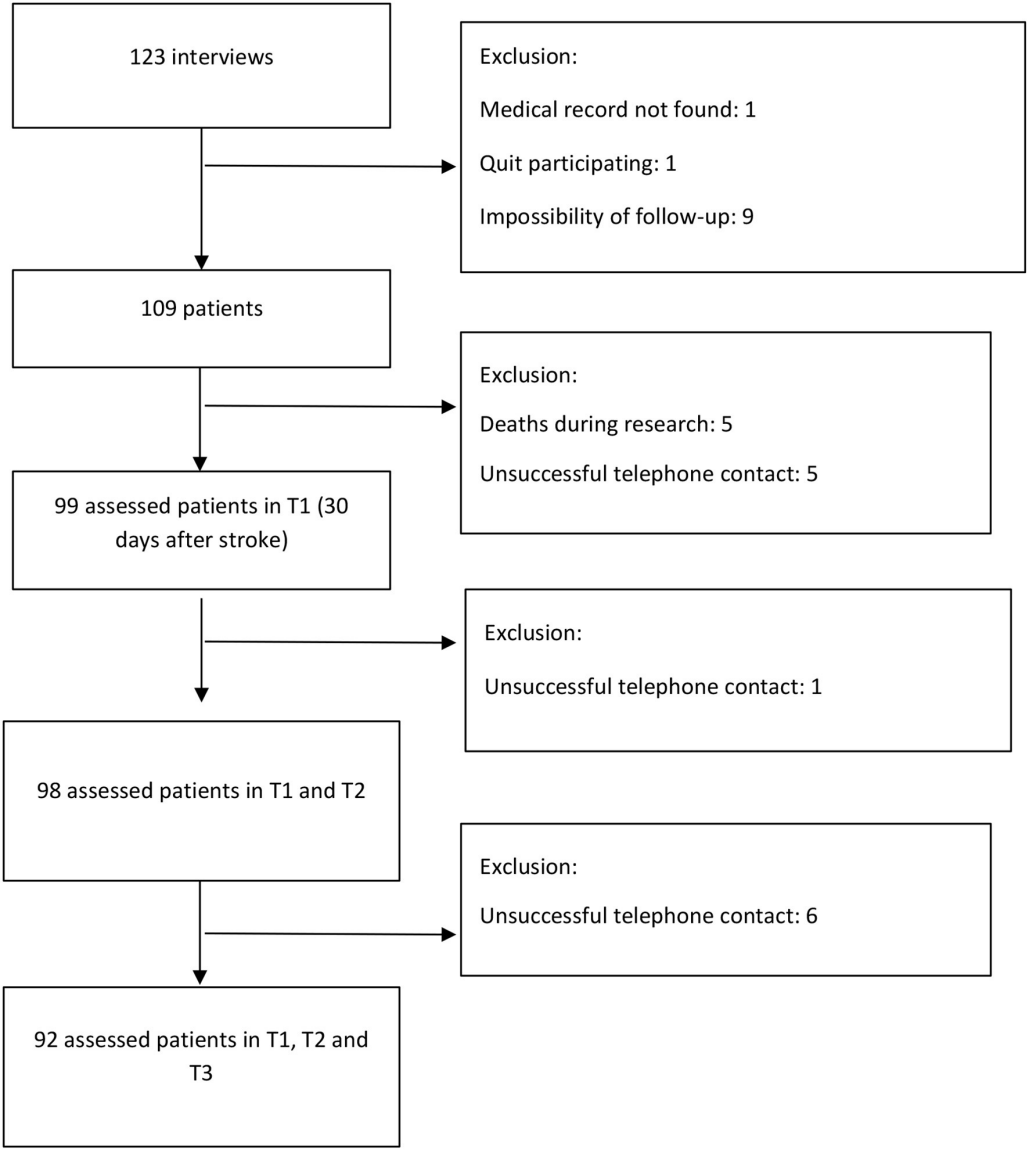
Flowchart of patient selection for the study.

We used software G ^*^ Power 3.1.9.2 to calculate sample size estimates. We adopted the objective of evaluating the correlation between the functionality of patients upon admission (modified Rankin Scale) and the quality of life and functional performance at three time points of follow-up (Barthel Index and EuroQol-5D). Considering a correlation of 0.30 (moderate) and a significance level of 1.67%, according to Bonferroni correction, the estimated sample was 112 patients, selected by convenience in consecutive order. Figure 1 shows the flowchart of participants' selection.

### Data collection

Several instruments were used to assess medical condition, functionality, and quality of life. Demographic information was obtained from the patients, and clinical information was obtained from the medical records. Data collection followed three methods:

**Individual interviews** with patients with stroke and/or their caregivers (T0). The duration of each interview ranged from 20 to 40 minutes. The researcher collected sociodemographic data (sex, age, education, occupation, and income), modifiable, non-modifiable risk factors, comorbidities (arterial hypertension, diabetes mellitus, heart failure, obesity, dyslipidemia, migraine, valve disease, atrial fibrillation, Chagas disease, among others), and the Frenchay Activities Index was applied to evaluate the performance of functional activities before the stroke.

**Inquiry in medical records** about the stroke' diagnosis (the number of events, signs, and symptoms), type (ischemic stroke—IS, hemorrhagic stroke—HS, transient ischemic attack—TIA), treatment performed, stroke severity measured by the National Institutes of Health Stroke Scale (NIHSS) upon admission and discharge, degree of disability assessed by modified Rankin Scale upon admission and discharge and assessment of the level of consciousness during admission using the Glasgow Coma Scale (GCS), length of stay, places of hospital stay (emergency department, critical care unit, and nursing unit), complications, use of antibiotics or vasoactive drugs, and performed tests.

**Telephone contact** was made after the patient had been discharged (T0), to assess the dependent variables (a measure of functionality and quality of life after the stroke), which were verified at three time points: 30 days (T1), 90 days (T2), and 180 days (T3) after stroke. The researcher read the scale items to all participants. Each interview ranged from 20 to 60 minutes as follows: (a) study presentation and explanation about the interview; (b) application of the scales EuroQol-5D and Barthel Index; and (c) conclusion and explanation of the possibility of new contacts.

### Measurements

National Institute of Health Stroke Scale (NIHSS)—Collected from medical records, the scale measures the severity and magnitude of neurological deficit after stroke on the level of consciousness, language, neglect, visual-field loss, extraocular movement, motor strength, coordination, dysarthria, and sensory loss through assessment of 15 items. The NIHSS score ranges from 0 to 42, with higher scores representing a more severe status (38).

Glasgow Coma Scale—Collected as a secondary source, it identifies neurological disorders and allows the evolution of the level of consciousness to be monitored and predict the prognosis. It ranges from 3 to 15 (39).

Modified Rankin Scale—Used in T0 at the time of subject inclusion in the study to assess the degree of disability after a stroke. The scale values range from 0 to 6 (38).

Frenchay Activities Index—The scale evaluates the performance of 15 instrumental activities in the last 3 or 6 months before the stroke: domestic activities, work/leisure, and outdoors. The score ranges from 0 (inactive) to 45 (very active). Values below 18 were considered predictors of mild disability after stroke (40).

EuroQol-5D—The scale assesses the quality of life, consisting of two components. The first is a descriptive system that defines the health-related quality of life (HR-QoL) in five dimensions (mobility, self-care, usual activities, pain/discomfort, and anxiety/depression) of three levels of severity each. Individuals can be classified into 243 different health states in a five-digit code (41, 42). The Total EuroQol Index, which corresponds to the “true” health status, is based on a formula in which each dimension has a different weight for assessing health. The scale score can vary from −0.59 to 1.00, with the highest score representing a better quality of life. The second component consists of the “Visual Analog Scale” (VAS) system, a scale numbered from 0 to 100. For the present study, only the first component was used in T1, T2, and T3, given the impossibility of assessing pain in all patients (41, 42).

Barthel Index — The scale evaluates the degree of independence in activities of daily living, including 10 personal activities: feeding, grooming, bathing, dressing, bowel and bladder care, toilet use, ambulation, transfers, and stair climbing. The score is calculated by summing the scores for each item. The total result is calculated by summing the scores for each item and it is always a multiple of 5. It ranges from 0 (completely dependent) to 100 (independent for basic ADLs). This scale was applied in time points 1, 2, and 3.

Glasgow Coma Scale (GCS) provides a practical method for assessment of impairment of the conscious level in response to defined stimuli, as sound or pressure. The GCS divides into three aspects of responsiveness: best eye response (E), best verbal response (V) and best motor response (M). The levels of response in the components of the Glasgow Coma Scale are ‘scored’ from 1, for no response, up to normal values of 4 (Eye-opening response) 5 (Verbal response) and 6 (Motor response). The total Coma Score thus has values between three and 15, three being the worst and 15 being the highest. The results of the GSC are used to guide early management of patients with a head injury or other kind of acute brain injury (39).

### Statistical analysis

The data were analyzed using the Statistical Analysis System (SAS), version 9.4. A 1.67% significance level was adopted for all tests (Bonferroni correction). Descriptive analyses described participants' demographic and disease characteristics. The Shapiro-Wilk test was used to check adherence to normal distribution.

The following tests were applied: Mann-Whitney non-parametric, Kruskal-Wallis tests, Pearson's chi-square test, Fisher's exact test, and the Spearman's correlation coefficient. The Cohen classification of this correlation was adopted: 0.1 to 0.29 (weak), 0.30 to 0.49 (moderate), and ≥0.50 (strong) (43).

Generalized linear models for multiple linear regression were used. Quality of life and functionality were considered dependent variables. Friedman's non-parametric test was used to compare quality of life and functionality scores between time points (T1, T2 e T3).

## Results

In total, 123 interviews were conducted. After drop-outs, we included data from 92 patients. These participants fulfilled their functional capacity and quality of life assessment by telephone contact 30, 90, and 180 days after stroke. Most patients were male (56.88%), with a mean age of 60.05 years (SD = 14.89), ranging from 22 to 91 years; 61.47% were married, 12.26% were illiterate, and 50% had incomplete elementary education. The average family income was USD 680.09 (SD = 457.63), 70.37% were professionally inactive at the time of the interview, and 17.59% were away from work due to limitations caused by the stroke.

Among the modifiable and non-modifiable risk factors for stroke, we identified systemic arterial hypertension (66.97%), previous or current smoking (62.62%), previous or current alcoholism (49.07%), physical inactivity (44.04%), dyslipidemia (41.28%), diabetes mellitus (33.94%), sleep disorder (25.7%), atrial fibrillation (16.51%), obesity (15.60%), and TIA (10.09%).

Regarding the type of stroke, 85.29% had an ischemic episode; 21% woke up with some degree of the deficit, 50.55% were home at the time of stroke, 56.99% were driven to the hospital by an ambulance, and 41.94% were driven in their own cars; 18.45% received thrombolysis, and 106 were discharged. The average length of hospital stay after stroke was of 9.9 days (SD = 12.73, 0–74).

Secondary data collection presented limitations due to the lack of standardized medical records. The GCS score upon hospital admission was recorded in 70 of the 109 medical records, with an average value of 13.60 points (SD = 2.70). This information was presented in 27 records at discharge, with an average value of 14.37 (SD = 1.52). We identified the NIHSS score upon admission in 72 of the 109 medical records, with an average of 8.29 (SD = 6.63). At discharge, registered in 57 records, this value decreased to 4.67 (SD = 5.27). mRS score upon admission was registered in 99 medical records, with an average value of 2.16 (SD = 1.78). At discharge, as registered in 49 medical records, the average value decreased to 1.88 (SD = 1.73).

The FAI showed an average of 25.07 (SD = 10.22). Assessed for all patients, the mRS functionality score during hospitalization was 2.24 (SD = 1.75). Assessed at three time points after the stroke, the BI functionality score ranged from 63.24 (SD = 35.16) at T1 to 77.23 (SD = 26.27) at T3. Over time, this difference was significant between T1 and T2, and T1 and T3. Regarding quality of life, we identified a significant difference between time points 1 and 3; i.e., quality of life went from an average of 0.35 (SD = 0.50) in T1 to 0.55 (SD = 0.42) in T3 (Table 1).

**Table 1:** Evolution of functionality and quality of life over time.

**Variable**	**Time**	**Mean**	**SD**	**Median**	*p*-value^*^
Barthel	T1^a.b^	63.42	35.16	75.00	**<0.0001**
	T2^a^	71.09	30.38	80.00	
	T3^b^	77.23	26.27	90.00	
EQ5D	T1^a^	0.35	0.50	0.37	**<0.0001**
	T2	0.46	0.44	0.59	
	T3^a^	0.55	0.42	0.70	

^*^Friedman' non-parametric test.

^a, b^Dunn-Bonferroni' post-test.

The bold numbers indicate a statistically significant correlation.

There was a positive correlation between functionality scores and GCS, and a negative correlation between NIHSS scores (upon admission and discharge) and functionality in all three time points. High NIHSS score was associated with low functionality. The correlation was considered moderate to high.

For length of stay, negative and moderate correlations were observed with length of stay in intensive care units (T2) and the wards (T1, T2, and T3). Functionality showed strong negative correlation with mRS score upon admission (Table 2).

**Table 2:** Spearman's correlation coefficient.

**Variables**	**Barthel**			**EQ-5D**		
	**T1**	**T2**	**T3**	**T1**	**T2**	**T3**
Age	−0.093	−0.082	−0.127	0.076	0.047	−0.020
GCS admission	**0.454** ^*^	**0.538** ^*^	**0.449** ^*^	**0.345** ^*^	**0.317** ^*^	0.284
NIHSS admission	–0.572^*^	–0.653^*^	–0.660^*^	–0.493^*^	–0.558^*^	–0.529^*^
NIHSS discharge	–0.584^*^	–0.572^*^	–0.457^*^	–0.409^*^	–0.462^*^	–0.448^*^
Length of stay	–0.349^*^	–0.415^*^	–0.417^*^	–0.395^*^	–0.416^*^	–0.380^*^
Length of stay at emergency	0.005	0.010	−0.051	−0.013	0.057	0.033
Length of stay at critical care unit	–0.299^*^	–0.339^*^	–0.291^*^	−0.250	–0.320^*^	−0.241
Length of stay at nursing unit	–0.343^*^	–0.407^*^	–0.389^*^	–0.412^*^	–0.432^*^	–0.398^*^
Rankin at hospitalization	–0.744^*^	–0.743^*^	–0.721^*^	–0.585^*^	–0.578^*^	–0.522^*^
FAI	−0.030	−0.009	0.053	0.017	0.042	0.042
Barthel	-	-	-	**0.734** ^*^	**0.753** ^*^	**0.700** ^*^

^*^p < 0.0125.

The bold numbers indicate a statistically significant correlation.

For quality of life, strong negative correlation with mRS score in all three time points and NIHSS upon admission in T2 and T3 was also seen; moderate negative correlation with NIHSS at discharge in all three time points was also seen with an extended general hospital stay (Table 2). Over time, there was a positive strong correlation between BI and EQ-5D in T1, T2, and T3 (Table 2).

Patients with comorbidities such as systemic arterial hypertension, atrial fibrillation, or patients receiving antibiotics, mechanical ventilation, oxygen therapy, enteral or bladder catheter, and Rankin score ≥2 showed less functionality and quality of life in all three time points (Table 3). We also found that the individuals who continued rehabilitation in the first 6 months (those with the most severe condition in this study) presented lower average BI and EQ-5D scores (Table 3).

**Table 3:** Comparison of means of functionality and quality of life.

**Variables**	**Categories**	**Barthel**			**EQ-5D**		
		**T1**	**T2**	**T3**	**T1**	**T2**	**T3**
Arterial hypertension	No	78.15	83.70	87.22	0.48	0.56	0.62
	Yes	57.31	65.85	73.08	0.30	0.41	0.52
		***p*** **=** 0.0063^*^	***p*** **=** 0.0018^*^	***p*** **=** 0.0036^*^	ns	ns	ns
Atrial fibrillation	No	69.22	77.01	83.05	0.42	0.53	0.61
	Yes	33.67	40.67	47.33	0.01	0.10	0.24
		***p*** **=** 0.0005^*^	***p*** **<** 0.0001^*^	***p*** **<** 0.0001^*^	***p*** **=** 0.0024^*^	***p*** **=** 0.0005^*^	***p*** **=** 0.0014^*^
Use of vasoactive drugs	No	73.18	78.27	83.27	0.47	0.55	0.63
	Yes	51.72	62.59	70.00	0.19	0.34	0.47
		***p*** **=** 0.0127^*^	***p*** **=** 0.0103^*^	ns	***p*** **=** 0.0139^*^	ns	ns
Use of antibiotics	No	74.29	81.03	84.44	0.46	0.56	0.63
	Yes	40.24	48.33	61.43	0.12	0.24	0.42
		***p*** **=** 0.0011^*^	***p*** **=** 0.0005^*^	***p*** **=** 0.0007^*^	***p*** **=** 0.0100^*^	***p*** **=** 0.0065^*^	ns
Use of mechanical ventilation	No	70.81	78.58	82.03	0.43	0.54	0.61
	Yes	28.50	30.50	54.00	−0.02	0.01	0.26
		***p*** **=** 0.0007^*^	***p*** **=** 0.0001^*^	***p*** **=** 0.0013^*^	***p*** **=** 0.0073^*^	***p*** **=** 0.0006^*^	***p*** **=** 0.0080^*^
Use of oxygen therapy	No	70.65	77.17	83.26	0.43	0.53	0.61
	Yes	43.33	53.00	57.67	0.13	0.25	0.40
		***p*** **=** 0.0035^*^	***p*** **=** 0.0029^*^	***p*** **=** 0.0021^*^	ns	ns	ns
Use of enteral catheter	No	79.10	84.51	87.21	0.51	0.57	0.63
	Yes	30.43	41.96	56.09	0.03	0.23	0.41
		***p*** **<** 0.0001^*^	***p*** **<** 0.0001^*^	***p*** **<** 0.0001^*^	***p*** **<** 0.0001^*^	***p*** **=** 0.0008^*^	***p*** **=** 0.0120^*^
Use of bladder catheter	No	75.55	81.33	85.70	0.47	0.56	0.63
	Yes	34.50	45.75	56.25	0.06	0.22	0.38
		***p*** **<** 0.0001^*^	***p*** **<** 0.0001^*^	***p*** **<** 0.0001^*^	***p*** **=** 0.0019^*^	***p*** **=** 0.0017^*^	***p*** **=** 0.0066^*^
	No	60.22	67.83	71.74	0.36	0.40	0.48
	Yes	70.49	77.07	84.76	0.40	0.55	0.65
		ns	ns	***p*** **=** 0.0111^*^	ns	ns	ns
	< 18	Angiotomography	77.37	80.26	0.40	0.46	0.61
	≥18		69.65	76.74	0.35	0.47	0.54
			ns	ns	ns	ns	ns
Rankin at hospitalization	< 2	FAI	87.00	92.09	0.56	0.64	0.71
	≥2		47.43	55.14	0.05	0.18	0.30
		***p*** **<** 0.0001^*^	***p*** **<** 0.0001^*^	***p*** **<** 0.0001^*^	***p*** **<** 0.0001^*^	***p*** **<** 0.0001^*^	***p*** **<** 0.0001^*^
Rehabilitation in T1	No	68.91	74.84	80.39	0.46	0.55	0.61
	Yes	52.04	62.22	70.00	0.13	0.25	0.43
		ns	ns	ns	***p*** **=** **0.0043**	***p*** **=** **0.0017**	ns
Rehabilitation in T2	No	70.00	75.00	80.34	0.47	0.55	0.63
	Yes	53.18	64.24	71.97	0.17	0.32	0.42
		ns	ns	ns	***p*** **=** **0.0093**	ns	ns
Rehabilitation in T3	No	72.35	78.37	83.57	0.54	0.60	0.68
	Yes	52.93	61.71	69.27	0.14	0.30	0.40
		***p*** **=** 0.0120^*^	***p*** **=** 0.0117^*^	***p*** **=** 0.0109^*^	***p*** **=** 0.0002^*^	***p*** **=** 0.0027^*^	***p*** **=** 0.0057^*^

ns, non-significant.

^*^Mann-Whitney.

The bold numbers indicate a statistically significant correlation.

For the functionality outcome, the regression model analysis revealed a negative relationship between age (T1 and T2), length of stay (T2 and T3), and Rankin score ≥2 (T1, T2, and T3). As for quality of life, a negative relationship to mRS score as only seen upon admission in T2, i.e., the EQ-5D score decreased by 0.76 with the 1-point increase in mRS score (Table 4).

**Table 4:** Multiple regression models.

**Independent variables**	**Dependent variable**	**Coefficient**	**95%CI**		***p*-value**	**Dependent variable**	**Coefficient**	**95%CI**		***p*-value**
			**LL**	**UL**				**LL**	**UL**	
Age (years)	Barthel T1	−0.01	−0.01	0.00	**0.0139**	EQ-5D T1	0.00	−0.02	0.02	0.9789
Length of stay (days)			−0.01	−0.02	0.00		0.0204		−0.04	−0.10	0.02	0.1757
FAI (≤18)			0.00	−0.01	0.00		0.3442		0.00	−0.02	0.02	0.8200
Sex (male)			0.01	−0.21	0.22		0.9491		0.15	−0.44	0.75	0.6121
Type of stroke (IS, TIA)			−0.12	−0.39	0.15		0.3971		0.13	−0.66	0.92	0.7409
Rehabilitation in T1 (no)			−0.04	−0.25	0.17		0.6949		−0.42	−1.10	0.27	0.2322
Rankin at hospitalization (≤2)			−0.59	−0.82	−0.35		**<0.0001**		−1.17	−2.21	−0.13	0.0269
Age (years)		Barthel T2	−0.01	−0.01	0.00		**0.0097**	EQ-5D T2	−0.01	−0.02	0.00	0.2830
Length of stay (days)				−0.02	−0.03		−0.01		**<0.0001**		−0.03	−0.06	0.00	0.0479
FAI (≤18)				0.00	−0.01		0.00		0.3958		0.00	−0.01	0.02	0.5931
Sex (male)			−0.06	−0.23	0.10	0.4565			0.13	−0.30	0.56	0.5485
Type of Stroke (IS, TIA)			−0.02	−0.22	0.18	0.8347			0.20	−0.39	0.78	0.5115
Rehabilitation in T1 (no)			0.02	−0.13	0.17	0.7670			−0.18	−0.64	0.28	0.4433
Rankin at hospitalization (≤2)			−0.42	−0.58	−0.26	**<0.0001**			−0.76	−1.31	−0.21	**0.0066**
Age (years)	Barthel T3		0.00	−0.01	0.00	0.0623	EQ-5D T3		−0.01	−0.01	0.00	0.1759
Length of stay (days)				−0.01	−0.01	0.00			**0.0011**		−0.02	−0.04	0.00	0.0207
FAI (≤18)				0.00	0.00	0.01			0.5679		0.00	−0.01	0.02	0.4928
Sex (male)			−0.02	−0.16	0.12	0.7857			0.05	−0.29	0.38	0.7832
Type of stroke (IS, TIA)			0.06	−0.09	0.20	0.4567			0.09	−0.31	0.49	0.6562
Rehabilitation in T1 (no)			−0.03	−0.13	0.08	0.6284			−0.19	−0.47	0.09	0.1730
Rankin at hospitalization (≤2)			−0.38	−0.51	−0.26	**<0.0001**			−0.35	−0.68	−0.03	0.0331

IS, ischemic stroke; TIA, transient ischemic attack; FAI, frenchay activity index; CI, confidence interval; LL: lower limit; UL, upper limit.

## Discussion

This study demonstrates there is a relationship between stroke patients' clinical and hospitalization data and functionality and quality of life in the first 6 months post-stroke. As expected, patients with more comorbidities or who presented more complications at hospitalization and therefore stayed for extended time at the hospital also presented worse functionality and quality of life after discharge at the three follow-up time point. In addition, improvement in functionality over time was seen to be related with an increase in quality of life. Further experimental research with multifactorial intervention during inpatient and home-based rehabilitation could focus on exploring and describing the effects of these interventions on quality of life and functionality.

The measure of functional independence at discharge is the strongest predictor at the 3-month time point, considering several predictive variables in other samples, including stroke-related comorbidities (44). These findings indicate that functional gains during hospital rehabilitation that enable independent living are sustained after 3 months.

Patients who underwent rehabilitation showed the most severe conditions and less quality of life. According to recommendations, early and multidisciplinary rehabilitation must be the first choice for patients affected by stroke, started at the hospital with follow-up treatment either as an outpatient or at home (45, 46). A lack of access to or delay in follow-up rehabilitation treatment for post-stroke people who are discharged and living at home can create barriers to their being able to return to everyday activities and community participation, and ultimately result in lower quality of life. However, we still observe this happening in developing countries, where rehabilitation services present deficiencies in meeting the heterogeneity of the stroke patients evaluated (47). In the study, even patients in the most severe conditions did not have rehabilitation started early.

According to the literature, functionality, and quality of life improve in the first six months after stroke. A study that assessed functional capacity with BI score of 152 in six months showed that approximately 30% of patients were functionally independent at the time of hospital discharge, and this number increased to 50% in the reassessment, with a decrease in patients with mild, moderate, severe, and total dependence (48). Another study, with 68 patients who underwent functional assessment simultaneously as in our study (30, 90, and 180 days after the stroke), also identified a progressive recovery pattern (49).

Quality of life is reduced in the period after the stroke, mainly due to lower psychological and spiritual wellbeing, the presence of depression, lower scores on the Barthel Index, or physical problems and incontinence (50, 51). For this reason, we think the quality of life assessment should be part of the formal monitoring of patients after stroke. This routine assessment can detect undiagnosed and potentially treatable complications, psychosocial problems, and neglected difficulties that often prevent the patient from regaining their functionality and wellbeing at its fullest.

Undoubtedly, the loss of functionality caused by a stroke significantly determines the loss of quality of life. Studies show that a significant portion of patients with stroke presented low scores on quality of life questionnaires that correlate with long-term moderate/severe disability values when assessed using scales such as BI or mRS (21). This suggests that medium- and long-term functionality can predict quality of life and social participation statuses (52).

However, even patients perceived as functionally independent still experience difficulties in social participation, depression, problems adapting to work, driving vehicles, new roles, and reintegration into society (53). In other words, even patients with average values on functionality scales report significant changes in their routine after a cerebrovascular event and difficulties in adapting to the new social roles.

In addition, individualized assessment of quality of life could become the target when designing a rehabilitation program (54). The information obtained in quality of life questionnaires can help develop more comprehensive rehabilitation nursing interventions and specific therapies. The routine post-stroke assessment could include quality of life measures to monitor rehabilitation programs focused on recovering functional and social capabilities.

Over the 6 months of follow-up, data showed that the improvement in functionality contributed to the improvement of QoL. Other studies present higher functionality at different times post-stroke. A study conducted in Singapore (35) reported mean functionality of 0.62 at 3 months post-stroke and mean functionality of 0.78 at 12 months post-stroke; another study in Germany (55) reported mean functionality of 0.81 at 2–3 years after the initial stroke. Other studies do not specify a time point post-stroke but report higher functionality values as well, such as in Korea (56) (mean = 0.76, no definition of time after stroke) and the United States (57) (mean = 0.78, no definition of time after stroke).

This study showed no correlation between the activities performed before the stroke as assessed by the FAI and quality of life and functionality after the 6 months of follow-up. This result is similar to a previous study (40), which presented a positive correlation between the FAI and functional capacity upon hospital admission and a negative correlation between the FAI and stroke severity. Additionally, a cohort study showed that the pre-stroke functional level predicts long-term survival after stroke (58).

Regarding sociodemographic data, age showed a negative relationship with functionality in time points T1 and T2. Still, other studies found that advanced age, lower socioeconomic status, and multiple comorbidities are negative predictive factors for late hospital rehabilitation (7 to 12 months after stroke) and functional independence 3 months after hospital discharge (44, 59).

Our sample's profile was similar to that of recent research (60, 61). When assessing the relationship between functionality and QoL with the sociodemographic data, comorbidities, and risk factors presented by the stroke patients, we found that older and less educated patients showed less favorable participation in daily-life activities up to 2 years post-stroke (62). Regarding stroke severity, a study showed that NIHSS upon admission predicts post-acute care disposition among stroke patients, a piece of information that may direct rehabilitation care (63).

### Limitations

The study was not without limitations. First, the study expected sample was not reached. It was considered that our results would present a moderate correlation between variables for sample estimation. However, a high magnitude of correlation was obtained between the variables, thus requiring a smaller sample size than we collected. It also missed secondary data from medical records, limiting our analysis possibilities. Despite the data collection team's training and supervision, this research is not free from observer and subject bias. Also follow-up evaluations were conducted only via telephone.

## Conclusion

There was a negative correlation between NIHSS and mRS scores and functionality and quality of life over a 6-month follow-up by telephone. This research found no relationship between activities performed before the stroke and functionalities and quality of life after the stroke. However, patients with comorbidities such as arterial hypertension, atrial fibrillation, bladder or enteral catheters, and extended hospital stay were associated with worse outcomes. In general, there was an improvement in BI and EQ-5D scores in the first 6 months of the stroke, and the increase in functionality contributes to an increase in QoL.

## Data availability statement

The original contributions presented in the study are included in the article/supplementary material, further inquiries can be directed to the corresponding author.

## Ethics statement

The studies involving human participants were reviewed and approved by the University of Campinas. The patients/participants provided their written informed consent to participate in this study. The studies followed the Declaration of Helsinki principles (64). Furthermore, this study was conducted following the guidelines, including informed consent and cooperation, voluntary participation, anonymity and confidentiality without affecting their rights to receive healthcare.

## Author contributions

Study conception and design, data collection, and data analysis and interpretation: AO-K and LB. Drafting of the article: AO-K, LB, and GS. Critical revision of the article: GS and LV. All authors contributed to the article and approved the submitted version.

## References

[1] Feigin VL Stark BA Johnson CO Roth GA Bisignano C Abady G. Global, regional, and national burden of stroke and its risk factors, 1990–2019: a systematic analysis for the global burden of disease study 2019. Lancet Neurol. (2021) 20:795–820. 10.1016/S1474-4422(21)00252-0 34487721 PMC8443449

[2] Oliveira GMMD Brant LCC Polanczyk CA Malta DC Biolo A Nascimento B. Cardiovascular Statistics–Brazil 2021. Arq Bras Cardiol. (2022) 118:115–373. 10.36660/abc.20211012 35195219 PMC8959063

[3] Silva S Rocha ECA Pontes-Neto OM Martins SO. Stroke care services in Brazil. J. Stroke Med. (2018) 1:51–4. 10.1177/2516608518776162

[4] Christensen MC Valiente R Sampaio Silva G. Acute treatment costs of stroke in Brazil. Neuroepidemiology. (2009) 32:142–9. 10.1159/000184747 19088487

[5] Alves MB Silva GS Miranda RCA. Patterns of care and temporal trends in ischemic stroke management: a Brazilian perspective. J Stroke Cerebrovasc Dis. (2017) 26:2256–63. 10.1016/j.jstrokecerebrovasdis.2017.05.008 28642017

[6] Rangel ES Belasco AG Dicinni S. Quality of life in patientes with stroke rehabilitation. Acta Paul Enferm. (2013) 26:205–12. 10.1590/S0103-21002013000200016

[7] Panício MI Mateus L Ricarte IF Figueiredo MM Fukuda TG Seixas JC. The influence of patient's knowledge about stroke in Brazil: a cross sectional study. Arq Neuropsiquiatr. (2014) 72:938–41. 10.1590/0004-282X20140167 25410321

[8] Chen Q Cao C Gong L Zhang Y. Health related quality of life in stroke patients and risk factors associated with patients for return to work. Medicine. (2019) 98:16. 10.1097/MD.0000000000015130 31008934 PMC6494282

[9] Mouelhi Y Jouve E Castelli C Gentile S. How is the minimal clinically important difference established in health-related quality of life instruments? Review of anchors and methods. Health Qual Life Outcom. (2020) 18:136. 10.1186/s12955-020-01344-w 32398083 PMC7218583

[10] WHOQoL Group. The World health organization quality of life assessment (WHOQoL): position paper from the world health organization. Soc Sci Med. (1995) 41:1403–10. 10.1016/0277-9536(95)00112-K 8560308

[11] Haas BK. A multidisciplinary concept analysis of quality of life. West J Nurs Res. (1999) 21:728–42. 10.1177/01939459922044153 11512210

[12] Bowling A. What things are important in people's lives? A survey of the public's judgements to inform scales of health related quality of life. SocSci Med. (1995) 41:1447–62. 10.1016/0277-9536(95)00113-L 8560313

[13] Zetola VF Rosa CT. Are we looking to stroke and quality of life? MOJ Gerontol Ger. (2019) 4:145–6. 10.15406/mojgg.2019.04.00196

[14] Katan M Luft A. Global burden of stroke. Semin Neurol. (2018) 38:208–11. 10.1055/s-0038-1649503 29791947

[15] Vercelli S Ferriero G Bravini E Al Yazeedi W Salgovic L Caligari M. A simple orthosis solves a problem in a patient with a dystonic finger after stroke. J Hand Ther. (2017) 30:113–5. 10.1016/j.jht.2016.04.003 27894678

[16] Froes KD Valdés MT Lopes DP Silva CE. Factors associated with health-related quality of life for adults with stroke sequelae. Arq Neuropsiquiatr. (2011) 69:371–6. 10.1590/S0004-282X2011000300020 21625768

[17] Xie J Wu EQ Zheng ZJ Croft JB Greenlund KJ Mensah GA. Impact of stroke on health-related quality of life in the noninstitutionalized population in the United States. Stroke. (2006) 37:2567–72. 10.1161/01.STR.0000240506.34616.10 16946158

[18] Ahlsio B Britton M Murray V Theorell T. Disablement and quality of life after stroke. Stroke. (1984) 15:886–90. 10.1161/01.STR.15.5.886 6236588

[19] Jonsson AC Lindgren I Hallstrom B Norrving B Lindgren A. Determinants of quality of life in stroke survivors and their informal caregivers. Stroke. (2005) 36:803–8. 10.1161/01.STR.0000160873.32791.20 15761203

[20] Cumming TB Brodtmann A Darby D Bernhardt J. The importance of cognition to quality of life after stroke. J Psychosom Res. (2014) 77:374–9. 10.1016/j.jpsychores.2014.08.009 25217449

[21] Patel MD McKevitt C Lawrence E Rudd AG Wolfe CD. Clinical determinants of long-term quality of life after stroke. Age Ageing. (2007) 36:316–22. 10.1093/ageing/afm014 17374601

[22] Naess H Waje-Andreassen U Thomassen L Nyland H Myhr KM. Health-related quality of life among young adults with ischemic stroke on long-term follow-up. Stroke. (2006) 37:1232–6. 10.1161/01.STR.0000217652.42273.02 16601213

[23] Clarke P Marshall V Black SE Colantonio A. Wellbeing after stroke in Canadian seniors: findings from the Canadian study of health and aging. Stroke. (2002) 33:1016–21. 10.1161/01.STR.0000013066.24300.F9 11935054

[24] Roding J Glader EL Malm J Lindstrom B. Life satisfaction in younger individuals after stroke: different predisposing factors among men and women. J Rehabil Med. (2010) 42:155–61. 10.2340/16501977-0497 20140412

[25] Palmcrantz S Holmqvist LW Sommerfeld DK. Long-term health states relevant to young persons with stroke living in the community in southern Stockholm: a study of self-rated disability and predicting factors. Disabil Rehabil. (2012) 34:817–23. 10.3109/09638288.2011.621507 22149134

[26] Cumming TB Churilov L Collier J Donnan G Ellery F. Early mobilization and quality of life after stroke findings from AVERT. Neurology^®^. (2019) 93:e717–28. 10.1212/WNL.0000000000007937 31350296 PMC6715509

[27] World Health Organization. How to use the ICF: A practical manual for using the International Classification of Functioning, Disability and Health (ICF). Exposure draft for comment. Geneva: WHO (2013). Available online at: http://www.who.int/classifications/drafticfpracticalmanual.pdf

[28] Mahoney FI Barthel DW. Functional evaluation: the Barthel index. Md State Med J. (1965) 14:61–5. 10.1037/t02366-000 14258950

[29] Moraes EN. Atençãoà and saúde do Idoso: Aspectos, Conceituais/Edgar Nunes de Moraes. Brasilia: Organização Pan-Americana da Saúde (2012), 98p.

[30] Baptiste S. Enabling communication in a person-centred, occupation-focussed context. In: eds Curtin M, Molineux M, Supyk J. Occupational Therapy and Physical Dysfunction: Enabling Occupation 6th ed. New York: Churchill Livingstone/Elsevier (2010) 2010:151–60.

[31] Serafin MB Ferreira TG Ponte AS Delboni MC. O conceito de autonomia sob a perspectiva de sujeitos acometidos por Acidente Vascular Cerebral. Revista Saúde. (2020) 46:1. 10.5902/2236583438723

[32] Burnagui JG Rosa MP Nascimento GCC. (2016). Autonomia e independência. Rev Ter Ocup Univ São Paulo. (2016) 27:21–8. 10.11606/issn.2238-6149.v27i1p21-28

[33] de Campos LM Martins BM Cabral NL Franco SC Pontes-Neto OM Mazin SC. How many patients become functionally dependent after a stroke? A 3-year population-based study in Joinville, Brazil. PLoS ONE. (2017) 12:e0170204. 10.1371/journal.pone.0170204 28107401 PMC5249115

[34] Virani SS Alonso A Benjamin EJ Bittencourt MS Callaway CW Carson AP. Heart disease and stroke statistics-−2020 update a report from the American heart association. Circulation. (2020) 141:e139–596. 10.1161/CIR.0000000000000757 31992061

[35] Yeoh YS Koh GC Tan CS Tu TM Singh R Chang HM. Health-related quality of life loss associated with first-time stroke. PLoS ONE. (2019) 14:e0211493. 10.1371/journal.pone.0211493 30689666 PMC6349359

[36] Carod-Artal FJ Trizotto DS Coral LF. Determinants of quality of life in Brazilian stroke survivors. J Neurol Sci. (2009) 284:63–8. 10.1016/j.jns.2009.04.008 (accessed July 25, 2022). 19411080

[37] Von Elm E Altman DG Egger M Pocock SJ Gøtzsche PC Vandenbroucke JP. The strengthening the reporting of observational studies in epidemiology (STROBE) statement: guidelines for reporting observational studies. Int J Surg. (2014) 12:1495–9. 10.1016/j.ijsu.2014.07.013 25046131

[38] Cincura C Pontes-Neto OM Neville IS Mendes HF Menezes DF Mariano DC. Validation of the national institutes of health stroke scale, modified rankin scale and barthel index in Brazil: the role of cultural adaptation and structured interviewing. Cerebrovasc Dis. (2009) 27:119–22. 10.1159/000177918 19039215 PMC7065394

[39] Jain S Iverson LM. Glasgow Coma Scale. In: StatPearls. Treasure Island (FL): StatPearls Publishing. (2022). Available online at: https://www.ncbi.nlm.nih.gov/books/NBK513298/ (accessed June 21, 2022).

[40] Monteiro M Maso I Sasaki AC Barreto N Oliveira J Pinto EB. Validation of the Frenchay activity index on stroke victims. Arquivos de neuro-psiquiatria. (2017) 75:167–71. 10.1590/0004-282x20170014 28355324

[41] Ferreira PL Ferreira LN Pereira LN. Contribution for the validation of the Portuguese version of EQ-5D. Acta Medica Portug. (2013) 26:664–75. 10.20344/amp.1317 24388252

[42] Pinto EB Maso I Vilela RN Santos LC Oliveira-Filho J. Validation of the EuroQol quality of life questionnaire on stroke victims. Arq Neuropsiquiatr. (2011) 69:320–3. 10.1590/S0004-282X2011000300010 21625758

[43] Cohen J. Statistical Power Analysis for the Behavioral Sciences. New Jersey: Lawrence Erlbaum, Associates (1988) 2:75–108.17695343

[44] Brown AW Lee M Lennon RJ Niewczyk PM. Functional performance and discharge setting predict outcomes 3 months after rehabilitation hospitalization for stroke. J Stroke Cerebrovasc Dis. (2020) 29:104746. 10.1016/j.jstrokecerebrovasdis.2020.104746 32151479

[45] Winstein CJ Stein J Arena R Bates B Cherney LR Cramer SC. Guidelines for adult stroke rehabilitation and recovery: a guideline for healthcare professionals from the American heart association/American stroke association. Stroke. (2016) 47:e98–e169. 10.1161/STR.0000000000000098 27145936

[46] Rasmussen RS Østergaard A Kjær P Skerris A Skou C Christoffersen J. Stroke rehabilitation at home before and after discharge reduced disability and improved quality of life: a randomised controlled trial. Clin Rehabil. (2016) 30:225–36. 10.1177/0269215515575165 25758941

[47] Della Vecchia C Préau M Haesebaert J Viprey M Rode G Termoz A. Factors associated with post-stroke social participation: a quantitative study based on the ICF framework. Annals Phys Rehabil Med. (2022) 3:101686. 10.1016/j.rehab.2022.101686 35779831

[48] López-Espuela F Pedrera-Zamorano JD Jiménez-Caballero PE Ramírez-Moreno JM Portilla-Cuenca JC Lavado-García JM. Functional status and disability in patients after acute stroke: a longitudinal study. Am J Crit Care. (2016) 25:144–51. 10.4037/ajcc2016215 26932916

[49] Kossi O Batcho CS Adoukonou T Thonnard JL. Functional recovery after stroke in Benin: a 6-month follow-up study. J Rehabil Med. (2016) 48:671–5. 10.2340/16501977-2128 27563697

[50] Chou CY. Determinants of the health-related quality of life for stroke survivors. J Stroke Cerebrovasc Dis. (2015) 24:655–62. 10.1016/j.jstrokecerebrovasdis.2014.10.022 25576350

[51] Wang R Langhammer B. Predictors of quality of life for chronic stroke survivors in relation to cultural differences: a literature review. Scand J Caring Sci. (2018) 32:502–14. 10.1111/scs.12533 28949412

[52] Tereza DM Baldasso GM Paes RS SÁ Junior AR Giehl MW Dutra RC. Stroke epidemiology in southern Brazil: Investigating the relationship between stroke severity, hospitalization costs, and health-related quality of life. An Acad Bras Cienc. (2022) 94:e20211492. 10.1590/0001-3765202220211492 35703701

[53] Sit JW Chair SY Choi KC Chan CW Lee DT Chan AW. Do empowered stroke patients perform better at self-management and functional recovery after a stroke? A randomized controlled trial. Clin Intervent Aging. (2016) 11:1441–50. 10.2147/CIA.S109560 27789938 PMC5072569

[54] Dabrowska-Bender M Milewska M Gołabek A Duda-Zalewska A Staniszewska A. The impact of ischemic cerebral stroke on the quality of life of patients based on clinical, social, and psychoemotional factors. J Stroke Cerebrovasc Dis. (2017) 26:101–7. 10.1016/j.jstrokecerebrovasdis.2016.08.036 27746082

[55] Lehnerer S Hotter B Padberg I Knispel P Remstedt D Liebenau A. Social work support and unmet social needs in life after stroke: a cross-sectional exploratory study. BMC Neurol. (2019) 19:220. 10.1186/s12883-019-1451-y 31492151 PMC6729017

[56] Kwon S Park JH Kim WS Han K Lee Y Paik NJ. Health-related quality of life and related factors in stroke survivors: data from Korea national health and nutrition examination survey (KNHANES) 2008 to 2014. PLoS ONE. (2018) 13:e0195713. 10.1371/journal.pone.0195713 29634768 PMC5892928

[57] Katzan IL Thompson NR Lapin B Uchino K. Added value of patient-reported outcome measures in stroke clinical practice. J Am Heart Assoc. (2017) 6:e005356. 10.1161/JAHA.116.005356 28733434 PMC5586276

[58] Chang WH Sohn MK Lee J Kim DY Lee SG Shin Y. Long-term functional outcomes of patients with very mild stroke: does a NIHSS score of 0 mean no disability? An interim analysis of the KOSCO study. Disabil Rehabil. (2017) 39:904–10. 10.3109/09638288.2016.1170214 27206550

[59] Yeh HJ Huang N Chou YJ Lee W Lai C Cheng C. Older Age, Low socioeconomic status, and multiple comorbidities lower the probability of receiving inpatient rehabilitation half a year after stroke. Arch Phys Med Rehabil. (2017) 98:707–15. 10.1016/j.apmr.2016.08.468 27633939

[60] Hubbard IJ Wass S Pepper E. Stroke in older survivors of ischemic stroke: standard care or something different? Geriatrics. (2017) 2:18. 10.3390/geriatrics2020018 31011028 PMC6371093

[61] Day CB Bierhals CC Mocellin D Predebon ML Santos NO Dal Pizzol F. Nursing home care intervention post-stroke (SHARE) 1 year effect on the burden of family caregivers for older adults in Brazil: a randomized controlled trial. Health Social Care Commun. (2021) 29:56–65. 10.1111/hsc.13068 32602588

[62] Verberne D Post M Köhler S Carey LM Visser-Meily J van Heugten CM. Course of social participation in the first 2 years after stroke and its associations with demographic and stroke-related factors. Neurorehabil Neural Repair. (2018) 32:821–33. 10.1177/1545968318796341 (accessed July 25, 2022) 30178696 PMC6146317

[63] Schlegel D Kolb SJ Luciano JM Tovar JM Cucchiara BL Liebeskind DS. Utility of the NIH Stroke Scale as a predictor of hospital disposition. Stroke. (2003) 34:134–7. 10.1161/01.STR.0000048217.44714.02 12511764

[64] World Medical Association. WMA Declaration of Helsinki–Ethical principles for medical research involving human subjects. (2018). Available online at: https://www.wma.net/policies-post/wma-declaration-of-helsinki-ethical-principles~-for-medical-research-involving-human-subjects/

